# The Political Determination of Gaza's Health System Destruction and Reconstruction and the Limitations of International Medical Deployments

**DOI:** 10.1002/hpm.70025

**Published:** 2025-09-22

**Authors:** Bilal Irfan, Ibrahim Omeish, Abdulwhhab Abu Alamrain, Belal Aldabbour, Arham Ali, Yassar Arain, Abeerah Muhammad, Khansa Irfan, Mohamed Fawaz, Yousef Barakat, Ruba Musallam, Osaama Khan, Karim Fikry, Muhammad Hamza Shah, Mohammed Halimy, Izzeddin Lulu, Tamer Alkurd, Hadeel Obeid, Roberto Sirvent, James Smith

**Affiliations:** ^1^ Center for Bioethics Harvard Medical School Boston Massachusetts USA; ^2^ Center for Surgery and Public Health Brigham & Women's Hospital Boston Massachusetts USA; ^3^ Department of Neurology University of Michigan Medical School Ann Arbor Michigan USA; ^4^ Department of Epidemiology University of Michigan School of Public Health Ann Arbor Michigan USA; ^5^ Harvard College Cambridge Massachusetts USA; ^6^ Faculty of Medicine Al‐Quds University Jerusalem Palestine; ^7^ Al‐Aqsa Martyrs Hospital Deir Al‐Balah Palestine; ^8^ Faculty of Medicine Islamic University of Gaza Gaza City Palestine; ^9^ Division of Pediatric Critical Care Department of Pediatrics Loma Linda University Children's Hospital Loma Linda California USA; ^10^ Department of Pediatrics Texas Christian University School of Medicine Fort Worth Texas USA; ^11^ Baylor Scott & White Health Dallas Texas USA; ^12^ University of Central Lancashire Preston UK; ^13^ University of Michigan Medical School Ann Arbor Michigan USA; ^14^ Intensive Care Unit Al‐Shifa Hospital Gaza City Palestine; ^15^ Northwestern University Feinberg School of Medicine Chicago Illinois USA; ^16^ Lahey Hospital and Medical Center Burlington Massachusetts USA; ^17^ School of Medicine Queen's University Belfast Belfast UK; ^18^ Al‐Shifa Hospital Gaza City Palestine; ^19^ Al‐Ahli Arab Hospital Gaza City Palestine; ^20^ Islamic University of Gaza Gaza City Palestine; ^21^ Department of Surgery Indonesian Hospital North Gaza Palestine; ^22^ Department of Nursing Indonesian Hospital North Gaza Palestine; ^23^ Department of Global Health and Social Medicine Harvard Medical School Boston Massachusetts USA; ^24^ Centre for Humanitarianism and Social Inclusion University College London London UK; ^25^ Medact London UK

**Keywords:** blockade, emergency medical teams, Gaza, health system, occupation, political determinants of health, reconstruction

## Abstract

Gaza's health system has been devastated by a confluence of political determinants that long predated the 2023–25 Israeli military assault and were dramatically intensified during it. Using historical, political economy, ethical, and health systems lenses, this article argues that settler colonialism, military occupation, and a protracted blockade created chronic shortages, workforce erosion, and institutional fragility, leaving services acutely vulnerable to targeted destruction of facilities and personnel. We examine the role and limitations of international medical deployments and field hospitals, which provided lifesaving care but operated under stringent access controls, supply interdictions, and security risks. Short rotations, poor continuity of care, and donor restrictions that discourage engagement with local authorities contributed to parallel systems, fragmentation, and dependency. We then identify four intersecting barriers to reconstruction: ongoing blockade and humanitarian access denials; lack of protection and accountability for attacks on health; governance fragmentation and the sidelining of Palestinian leadership; and donor fatigue amid politicised aid. The article proposes a justice‐centred pathway for recovery that prioritises accountability and reparations, an end to the blockade and occupation, inclusive Palestinian‐led governance, alignment of aid with national plans, avoidance of parallel structures through early transition to local ownership, workforce stabilisation, and long‐term partnerships. Without these political preconditions, reconstruction efforts will remain fragile and inequitable.

## Highlights

- Gaza's health collapse is politically produced, not collateral.
- International deployments give relief but entrench parallel systems.
- Blockade, access denials, and impunity obstruct reconstruction.
- Recovery needs accountability, de‐occupation, and Palestinian leadership.

## 1 Introduction

The current catastrophic state of health and the threat to survival for Palestinians in Gaza is inextricably connected to a series of foundational political determinants and structural drivers [1, 2, 3]. Amid what leading human rights and humanitarian organisations, United Nations (UN) independent experts, and lawyers and scholars in relevant disciplines, have characterised as genocide, perpetrated by Israel against Palestinians living in Gaza, the enclave's already fragile health system has been repeatedly and systematically targeted to the point of collapse [4, 5, 6, 7, 8, 9, 10, 11, 12]. Israeli forces have levelled hospitals and clinics, killed, injured, and detained thousands of medical personnel, and repeatedly denied the entry of essential medical supplies and personnel. These actions have been carried out against the backdrop of clear genocidal intent to destroy in whole or in part the Palestinian people in Gaza [9, 13, 14, 15]. This unprecedented assault on health cannot be dismissed as the unavoidable collateral damage of war, but rather is the predictable result of long‐standing political conditions ‐ settler colonialism, occupation, nearly 2 decades of blockade, and a wider climate of impunity, collusion and indifference ‐ that have rendered Gaza's healthcare system‐ and all Palestinians in Gaza ‐ extraordinarily vulnerable amid Israel's current assault [16].

It is within this context that a constellation of emergency response‐oriented medical teams have been deployed to Palestine ‐ and predominantly Gaza ‐ to provide care. Many of these medical teams have been organised under the umbrella of the World Health Organisation's (WHO) emergency medical teams (EMT) initiative. While these deployments are intended to provide short‐term relief, and involve several commendable individual and collective efforts, they often operate within—and frequently reinforce ‐ the structural constraints imposed by the overarching conditions of Israel's settler colonial occupation.

This paper examines how political determinants ‐ the political structures and resulting policies and practices that shape what manifests most immediately as a catastrophic humanitarian crisis in Gaza ‐ have functioned to obscure avenues for the justice‐centred reconstruction and recovery of Gaza's health system, and how international medical deployments have grappled with these constraints. Recent events throughout the course of Israel's ongoing genocide, including the targeted killing of aid workers, the withdrawal of international teams, and the emergence of parallel, internationally‐run field hospitals, showcase the urgent need to critically assess the limitations of dominant forms of contemporary medical humanitarian response in Gaza. By engaging with the political economy of health service provision amid the ongoing genocide, we employ historical, political, ethical, and health systems lenses to argue that without accountability for the violence and destruction committed by Israel, coupled with the reform of hegemonic humanitarian and development approaches, efforts to rebuild Gaza's health system will be frustrated, remain fragile, and only deepen existing structural injustice [17].

## 2 Pre‐2023 Structural Fragility Under Occupation

Prior to the precipitous escalation in violence since October 2023, Gaza's health system had been debilitated by decades of blockade, military occupation, recurrent Israeli military assaults, and political division [18]. Israel's imposition of a total land, sea, and air blockade on Gaza since 2007 had led to chronic shortages of essential medicines, equipment, and trained personnel [19]. The deliberate, systematic, and targeted imposition of restrictive and obstructive measures has resulted in the collective deprivation of the right to the highest attainable standard of health for Palestinians living in Gaza, in clear violation of international humanitarian law [20, 21, 22]. Vital proximate determinants of health, including access to clean water and comprehensive sanitation services along with the reliable supply of electricity, have been repeatedly compromised over almost 2 decades [16].

By 2023, hospitals relied on precarious backup generators due to daily power outages, while over one third of essential drugs were routinely at zero stock [23, 24, 25]. The split between Palestinian governance structures in Gaza and the West Bank has served to further jeopardise health services, as thousands of public employees working within Gaza's health sector were unpaid or underpaid, ministries were duplicated and underfunded, and clear lines of authority and oversight were absent [26]. International donor policies deepened these structural constraints as many key donors cut or conditioned funding to Gaza following the imposition of the Israeli blockade, channeling aid to NGOs or Palestinian Authority (PA)‐linked facilities rather than to areas where assistance was most needed, or to Palestinian institutions best placed to deliver services [26]. Counter‐terrorism legislation and strict ‘no‐contact’ rules enforced by influential European and North American governments effectively meant that Gaza's Ministry of Health (MOH) received little direct external support [27, 28]. These highly restrictive external foreign policy decisions, wherein Gaza's health authority was largely excluded, have served to accelerate the fragmentation of the health system and the NGOization and privatisation of essential service provision, for which parallel providers (UNRWA for refugees, numerous NGO clinics, and private facilities) attempted to fill growing gaps.

On the eve of Israel's genocidal escalation in 2023, Gaza's healthcare system had been overstretched and left critically under‐resourced as a result of nearly 2 decades of blockade, chronic underinvestment, and repeated Israeli military assaults. Though the health system had demonstrated adaptability during previous Israeli assaults (e.g., 2008–09, 2012, 2014, 2021, and other attacks), each escalation left the health system more debilitated and less able to meet the growing health needs of the population. Public hospitals lacked the ability to absorb shocks, and were made heavily dependent on external support by design [17, 29, 30]. Additionally, repeated assaults and low salaries have triggered emigration, with many skilled healthcare workers seeking safety and better‐paying jobs abroad, inadvertently depriving communities in Palestine of their expertise. Meanwhile, the combined effects of chronic fiscal deficits and Israeli restrictions on the entry of various materials and advanced medical devices created a gap that had to be filled by medical referrals to other parts of Israeli‐occupied Palestine, generating even greater dependence on the Israeli security apparatus, and leaving the population vulnerable to ever‐changing criteria, and the repeated, arbitrary denial of travel permits [31].

Subsequently, the baseline health of the Palestinian population in Gaza was precarious, with rising rates of micronutrient deficiencies and trauma‐related disability affecting all age groups [32, 33, 34, 35]. Even before the latest escalation, under‐five mortality in Gaza was approximately five times higher than in Israel, while maternal mortality was approximately six to eight times higher, reflecting clear structural inequalities in health outcomes [36, 37, 38, 39]. In short, the crisis affecting the health sector was political in origin: a product of settler colonialism, and the cumulative consequence of Israel's occupation and its intentional and totalising blockade, underfunding tied to the wider geopolitical allegiances of Israel's allies, and the absence of sovereign control over either the health system or the underlying determinants of health. These factors coalesced to establish the brittle foundation upon which Israel's firestorm in late 2023 was unleashed.

## 3 Israel's Destruction of Gaza's Health Infrastructure (2023–Present)

Israel's military assault on Gaza, which escalated in October 2023, pushed an already strained health system to a state of total collapse [40]. Sustained Israeli air and artillery bombardment and the Israeli ground invasion targeted medical facilities and healthcare workers (HCWs) on an unprecedented scale, in flagrant violation of international humanitarian law [9, 41]. As of late 2024, Medecins Sans Frontieres (MSF) reported that Gaza's healthcare system lay in ruins, with ongoing Israeli military attacks and a protracted siege functioning to destroy the conditions of life [15]. By early 2025, almost all of Gaza's hospitals and clinics had been damaged, destroyed, or left minimally operational, including major referral centres [13, 17]. The Al‐Shifa Medical Complex, Gaza's largest tertiary hospital, was extensively damaged after a protracted siege and successive raids by the Israeli military that killed patients and staff, drawing only perfunctory international outcry [42]. Gaza's only oncology centre was rendered out of service due to direct damage and direct occupation by the Israeli military, further imperilling some 10,000 cancer patients [43, 44, 45]. Maternity hospitals suffered too; Al‐Hilal Maternity Hospital in Rafah, and Kamal Adwan Hospital in northern Gaza, were besieged for months in 2024, and for the latter again in 2025, while the maternity hospital that was part of the Al‐Shifa Medical Complex was forced out of service at one point [46, 47]. As a result, women have been forced to give birth in tents, field hospitals, smaller private clinics, or make‐shift camps, often without access to basic medical supplies or anaesthesia [48, 49].

HCWs have also become targets [50, 51]. Palestinian and international organisations have documented relentless assaults on ambulance crews, doctors, nurses, and other healthcare professionals [52]. In the first three months of the genocide, hundreds of Palestinian HCWs were killed—an estimated 480 by mid‐2024, alongside a further 160 detained or missing [43]. By mid 2025, it was estimated that approximately 1600 Palestinian HCWs have been killed by the Israeli military, far overshadowing the number of HCWs killed in other recent armed conflicts [53]. A staggering number of UN personnel have been killed; as of May 2025, at least 300 UNRWA staff—including doctors, nurses, and teachers—had been killed in Gaza [54, 55]. UN facilities, which are always clearly marked and for which coordinates are routinely shared with the Israeli military, have not been spared from attacks; UN shelters and UN schools‐turned‐displacement‐shelters were repeatedly struck by Israel, prompting the UN Secretary General to condemn attacks against UN premises [54]. Such direct and indiscriminate violence against health and humanitarian workers, and the systematic nature of both destruction and obstruction—hospitals reduced to rubble, rescue teams prevented from reaching the wounded, supply convoys prevented from reaching affected communities, entire residential neighborhoods flattened—supports the assessment by numerous human rights and humanitarian organisations and legal and other disciplinary experts that Israel's violence has functioned to physically destroy in whole or in part the Palestinian people in Gaza [56].

The result for health specifically is a scattered and heavily compromised health system [57]. The remaining partially operational health facilities are overwhelmed, and all lack sufficient electricity, clean water, fuel, oxygen, and medical supplies. In the spring of 2025, the Al‐Ahli Arab Baptist Hospital's oxygen units and building had been struck by the Israeli military, while both the European Gaza Hospital (EGH) and Nasser Medical Complex were attacked [58, 59, 60, 61, 62, 63]. At the same time, bed occupancy has surged far beyond 100% in several hospitals, while surgeons have been forced to perform amputations and cesarean sections by the light of mobile phones, often without adequate anaesthesia or oxygen [64, 65, 66]. Communicable disease outbreaks (e.g., respiratory and water‐borne infections, scabies, etc.) have proliferated among displaced people who are confined to overcrowded shelters, while routine immunisation services and continuity of chronic disease care have been heavily compromised [67, 68, 69, 70, 71]. In short, Israel's genocidal violence in Gaza has not only directly killed tens of thousands of Palestinians, but it has also destroyed the very institutions and people that are meant to administer care, safeguard health, and sustain life [72].

## 4 Limitations of International Medical Deployments: Short‐Term Interventions and Parallel Systems

In response to Israel's escalating violence in Gaza, and its immediate humanitarian and health consequences, a multitude of international medical and humanitarian deployments were organised after October 2023 by several NGOs (e.g., Médecins Sans Frontières, Medical Aid for Palestinians, the Palestine Children's Relief Fund, among many others), the International Committee of the Red Cross (ICRC) and national Red Cross and Red Crescent societies, UN agencies, foreign governmental initiatives, and faith‐based charities. Field hospitals and emergency clinics sprouted in the less‐heavily targeted areas of Gaza, largely in Al‐Mawasi in the south, and near Khan Younis and Rafah, where many displaced HCWs had also been forced to relocate. For example, the International Medical Corps (IMC) deployed a large field hospital in early 2024, providing trauma surgery and primary care to trauma‐wounded and other conflict‐affected people [73]. The hospital was first located between Rafah and Khan Younis, before teams relocated to Al‐Mawasi as attacks on Khan Younis escalated in the early months of 2024. The ICRC, in coordination with the Palestine Red Crescent Society (PRCS), opened a 60‐bed field hospital in response to growing trauma‐related needs due to repeated mass casualty incidents and mounting pressure on the few remaining public hospitals. Egypt, Jordan, and the UAE, as well as global Islamic charities, established makeshift surgical units and clinics near the Rafah border (before Israel's ground invasion of Rafah in May 2024), and treated thousands of patients who could not access health facilities elsewhere [73, 74]. Even the Israeli military publicised its own ostensible humanitarian efforts, claiming to facilitate some field hospitals and medical corridors given that Israel as the occupying power exerts control over any aid entering Gaza [73].

International healthcare workers were able to provide medical care under extraordinarily difficult conditions, though by virtue of their nationalities and institutional affiliations were generally spared exposure to the full extent of violence and deprivation inflicted on their Palestinian counterparts. In many cases, they became the only source of certain medical services as nearby MOH facilities were targeted, besieged, raided, occupied, destroyed or otherwise rendered unusable or ineffective. For example, MSF teams set up surgical suites in whatever safe structures could be found, performing emergency amputations and treating shrapnel wounds amid artillery fire and airstrikes [75, 76, 77, 78]. Palestinian staff were joined by international HCWs to triage and treat patients arriving during mass casualty incidents from near‐continuous airstrikes [79]. In less overwhelming moments, international HCWs were able to briefly relieve their Palestinian counterparts from duty, providing them with time to sleep, visit their families, and fulfil other needs such as searching for water, food, and other basic necessities [80]. Telemedicine networks sprang up to provide remote guidance for complex surgical care at a time when Palestinian specialists had been killed or detained, and international specialists could not enter Gaza [81]. Similarly, staff from UNRWA (now designated a terrorist organisation in an unprecedented move by the Israeli Knesset), the Palestine Medical Relief Society, and other Palestinian NGOs managed primary healthcare for hundreds of thousands of displaced people, at times operating out of school basements and tent clinics [82].

The short‐term relief provided by deployments of international healthcare workers undoubtedly achieved some positive impact at a time of profound manufactured scarcity and near‐overwhelming medical needs. However, during the first months of the genocide there were signs that these efforts were profoundly constrained: a band aid on a gaping wound. All international humanitarian programs in Gaza remain subject to political gatekeeping, overwhelmingly by Israel as the occupying power, which actively exerts complete control over the armistice line crossings, and now the southern border with Egypt. Periodic so‐called ‘humanitarian pauses’ have been needed simply to move convoys containing medical and surgical supplies and international medical teams within different parts of Gaza and at times through the Rafah crossing [83, 84, 85, 86, 87]. The term ‘humanitarian pause’ is tellingly euphemistic; it was deliberately adopted by political leadership seeking to avoid the use of the term ‘ceasefire’, thus signalling a conscious disregard for a comprehensive cessation of hostilities.

Even then, what trickled through was woefully insufficient, prompting UN agencies to repeatedly decry that aid convoys were ‘a drop in the ocean’ and that medical teams lacked safety guarantees amid the ongoing bombardment [88, 89]. The invasion, occupation and destruction of the Rafah crossing by the Israeli military in May 2024 precipitated even greater restrictions on humanitarian access, as well as an almost complete cessation of medical evacuations out of Gaza for a period of several months. Within the first month following the destruction of the Rafah crossing, there was a 67% drop in the number of aid trucks entering Gaza, exacerbating an already dire shortage of food and specialist nutritional supplements, medical supplies, fuel, and many other essential commodities [90].

In addition to growing restrictions on humanitarian access, 44% of international HCWs registered under the WHO EMT initiative who have attempted to enter Gaza have been denied entry since the breakdown of the ceasefire in March 2025 [91, 92]. This has only compounded staffing shortages ‐ specifically concerning certain surgical specialties ‐ within an overstretched and exhausted healthcare workforce. EMTs that are approved entry are subjected to heavy restrictions by the Israeli military: two bags of personal items only, and no medical supplies. On occasion baby formula, personal food supplies, and medications as simple as paracetamol, have been confiscated by Israel at the Allenby crossing in the occupied West Bank. The inability of medical teams to carry medical supplies renders their presence markedly less useful, where the intentional deprivation of basic diagnostic tools and treatments continues to compromise clinical care.

In March 2025, Red Crescent paramedics and other humanitarian workers were abducted, handcuffed, or killed in sustained live fire [52, 93]. The volatility of Israel's ongoing assault has meant that medical teams could be forced to evacuate at any moment, as seen in the case of Israel's ground offencive on Rafah in May 2024, in several hospitals in Gaza's northern governorate in late 2024, and the European Gaza Hospital in May 2025.

Many of the international staff working with international NGOs were evacuated in the first weeks of the genocide due to security concerns, leaving programs to be managed by Palestinian staff who faced a constant and ever‐growing threat of violence. Throughout 2024, as Israel expanded its offencive to southern Gaza, humanitarian organisations acknowledged a reality familiar to all Palestinians in Gaza: that nowhere was safe [94]. This extended to supposedly protected spaces, such as hospitals, prompting many organisations to repeatedly demand protection for HCWs and the safeguarding of health facilities [95, 96]. These warnings proved prescient, as marked aid vehicles and well‐known aid locations came under repeated direct fire. One such example of the persistent threat of violence against health and humanitarian workers came in April and November 2024 with targeted Israeli strikes on the humanitarian organisation World Central Kitchen (WCK). WCK, an international NGO providing meals in many countries affected by humanitarian crises, had established programs in Gaza to feed displaced people and hospital patients. On April 1, 2024, an Israeli drone strike repeatedly hit a convoy of clearly marked WCK vehicles in southern Gaza, killing seven WCK team members who had been hired to support the delivery of food [97]. In November 2024, another WCK vehicle was struck from the air in Khan Younis, killing three more WCK staff [98]. The immediate impact was chilling, as WCK announced its intention to pause all of its programs in Gaza following these incidents. This meant the loss of a vital food lifeline for both patients and displaced people, showcasing how targeted violence can abruptly disrupt access to essential humanitarian services. Similar dynamics have played out with medical teams. By late 2024, many international NGOs had withdrawn or drastically scaled‐down their international staff presence due to security concerns, essentially leaving the health response again to Palestinian HCWs and a small number of higher‐risk international deployments. At least five of MSF's Palestinian staff in Gaza were killed in the initial months, and the organisation repeatedly protested the bombing of hospitals that it supports, at times halting operations when forced displacement orders issued by the Israeli military or attacks rendered the continuation of medical activities impossible [75, 99, 100]. After dozens of its shelters were hit, the UN relocated many of its international staff, and continued to operate largely through its Palestinian teams who have remained in Gaza. As aid workers have been killed or injured ‐ be they UN‐affiliated doctors or Red Crescent paramedics ‐ international NGOs have continually reassessed the risk of existing or future deployments. By early 2025, the cumulative effect of these targeted killings was a substantial scaling down of international medical and humanitarian workers in Gaza, dramatically compounded by Israel's escalating denial of access for those who still sought to enter Gaza [101]. What remained was a very small international HCW presence, with Palestinian HCWs holding together basic service provision for a population of over 2.1 million people confined to an ever‐shrinking proportion of the enclave.

International medical deployments during Israel's genocide in Gaza have played an important role, yet they also serve to illustrate the inherent limitations wherein humanitarian response is used as a substitute for a political action and the pursuit of just resolution. Several critiques emerge from recent experiences and the existing literature related to humanitarian health interventions in contexts affected by armed conflict.

### 4.1 Short‐Term Relief versus Long‐Term Capacity

By design, short‐term medical deployments and field hospitals are intended to provide short‐term relief. They are oriented towards surge support to save lives in the emergency phase of a crisis, managing trauma, stemming disease outbreaks, and supplementing existing capacity to manage overwhelming health needs in contexts affected by humanitarian crises. What they are not designed to deliver is a sustainable health response. In Gaza, as past violent escalations have shown, once the emergency phase subsides, these international medical deployments pack up or scale down. Even within the emergency phase, there is typically a lack of coordination between outgoing and incoming EMTs from different NGOs, which can have a significant impact on continuity of care for patients. Additionally, due to the short two to 4 week duration of these medical deployments, patients are not always able to complete treatment administered by international specialists. Nasari et al. observed that ‘temporary emergency aid cannot compensate for the structural weaknesses that have long defined Gaza's health sector’ [17]. Indeed, decades of such missions to Gaza (and similar settings) have not prevented the decline of the health system. A stark example is the case of prosthetic care: multiple international medical teams have entered Gaza to perform amputations or deliver prosthetic limbs to people maimed in military attacks, but if local rehabilitation facilities and supply chains are not rebuilt, the follow‐up care for those patients quickly falters [102]. Palestinian health workers often express a sense of being left to pick up the pieces with scant resources once media attention shifts, while they are often left with mismatched medical supplies that may be incompatible with existing equipment and training and therefore unusable [103]. This inconsistency in medical supplies complicates the pursuit of standardised care pathways or the consistent implementation of clinical guidelines and protocols that can improve health outcomes. The episodic nature of violence has repeatedly demonstrated this pattern in Gaza; after Israel's 2014 assault many international medical teams left within months, while Gaza's own hospitals struggled for years to cope with unmet surgical and mental health needs [104]. The fundamental limitation is that short term medical deployments shaped by modalities of humanitarian government fail to adequately recognise or engage with political root causes, and may even function to normalise said root causes [105]. There is a well documented tendency that humanitarian interventions serve as a fig leaf for political inaction, allowing the violence of the occupation and blockade to continue behind a facade of humanitarian concern [106, 107, 108].

### 4.2 Parallel Systems and Fragmentation

A recurring critique in humanitarian health responses is the tendency towards the creation of parallel health systems. In Gaza, this phenomenon has become more evident since late 2023. With international‐run field hospitals and clinics, there is concern that a parallel structure emerges alongside ‐ or in place of ‐ the Indigenous health system. For example, the large UAE‐funded field hospital in Rafah, which by late 2024 had reportedly treated nearly 50,000 patients, operates under Emirati management with its own supply chain and staff. While undeniably providing important life‐saving services, such a facility is not fully integrated into Gaza's domestic health system and governance structures. As Ministry of Health facilities have been targeted and attacked, and healthcare workers in the public sector have gone without pay for protracted periods of time, such facilities have drawn healthcare workers away with higher salaries, and attracted donor funding that might otherwise strengthen local facilities. The WHO and humanitarian experts have long warned that creating duplicate systems should be a last resort, to be avoided unless absolutely necessary [109]. In protracted crises such as Syria, and now Gaza, multiple health providers offer services in parallel: one managed by the health authorities, another by UNRWA for refugees, and others by Palestinian and international NGOs. This fragmentation can reduce overall effectiveness and introduce inefficiencies. Patients might not know where to seek a particular service, while records and referral pathways may not be shared between providers. Moreover, when certain service providers eventually withdraw, without proper handover and the integration of essential services, affected communities are left with a weakened healthcare system. In the case of Gaza, the risk is that reliance on internationally‐run field hospitals and vertical programs (e.g., an internationally‐run clinic for specific medical problems) will undermine the reestablishment of Gaza's domestic healthcare delivery capacity. Ideally international assistance should work to reinforce the national health system, not supplant it. Yet overarching political conditions complicate this as donors refuse to work directly with the Ministry of Health, choosing to circumvent its capacity and expertise [110]. The result is a patchwork health sector and the diminishing prospect of cohesive health system recovery [36].

### 4.3 Lack of Coordination With Palestinian Authorities

Closely related is the reluctance of many international medical teams to engage with Gaza's health authorities [111]. Experienced humanitarian organisations strive to coordinate with local health officials even in difficult political contexts, recognising that local knowledge, expertise, and oversight are key [112]. In Gaza, coordination occurs at the technical level, though even this is limited. For example, Palestinian hospital directors guide international surgical teams to where they are needed most urgently, but this in turn may be limited to a specific governorate [113]. Institutionalised partnerships are largely absent, as donor restrictions preclude many NGOs from formal collaboration with the Palestinian authorities in Gaza [111]. The local authorities are almost always those who have the most comprehensive understanding of population health needs, and who will remain long after international organisations leave. The disconnect regularly leads to misaligned priorities: an international NGO might open a trauma clinic in a particular location without realising that the Ministry of Health had already planned such a project, or conversely multiple NGOs might cluster around certain hospitals (where access is easier) while other facilities receive little or no support [113]. OCHA has documented cases of ‘duplication and omissions’ in service delivery in such environments [114]. In late 2023, an effort by Health Cluster partners was made to establish a centralised coordination mechanism for the health response, but without strong involvement from the Ministry of Health this also risked the imposition of an externally‐imposed parallel structure. This limitation is manufactured and fundamentally political, insofar as it reflects the ambivalence of several governments to engage with Palestinian organisations and Gaza's authorities. A truly patient‐centred approach would press international actors to work with whoever can deliver care most effectively with a commitment to needs‐based programming. Some NGOs (e.g., MSF, ICRC) coordinate better with Gaza's health authorities, demonstrating the extent to which political distance is a choice, often related to a lack of institutional financial independence [112, 115].

### 4.4 Reinforcement of Dependency

The proliferation of international medical teams and programs ultimately function to reinforce Gaza's dependency on external aid, with few exceptions [113]. Before the genocide, Gaza was often cited as an aid‐dependent society with a fragile economy, heavily affected by Israel's strategy of ‘de‐development’ [113]. The health sector exemplified this state: even routine tertiary care like cancer treatment often depended on patient transfers out of Gaza or the donations of drugs [116, 117]. Israel's genocide has exacerbated this dependence to extreme levels, driving a reliance on external support for access to critical resources, from dialysis solutions to specialist trauma surgeons [118]. International medical deployments can fulfil a service delivery function that the local health system would ordinarily provide. For example, when war amputees require physical rehabilitation ‐ an essential service that should ordinarily be delivered by a fully functioning local health system ‐ Palestinians in Gaza have been forced to depend on rotating teams of international volunteers who often participate in transient deployments. Although these interventions provide short‐term relief, they do little to cultivate a sustainable local rehabilitation workforce or corresponding infrastructure. The availability of temporary external substitutes can diminish donors' incentives to invest in durable, locally anchored rehabilitation capacity. It is a well‐known paradox in humanitarian programming that prolonged, large‐scale direct service provision by external actors typically impedes the revival of local systems. In Gaza, therefore, any internationally managed service ‐ such as the network of NGO‐led field hospitals ‐ that remains active into the reconstruction phase should articulate a clear strategy for the transfer of oversight to the local health ministry and training for local staff to sustain service provision. Unsurprisingly, Palestinian clinicians and health‐sector planners have often voiced frustration that, despite the presence of many NGOs, they observe little net improvement in their own institutions [119].

### 4.5 Ethical Implications

One must not overlook the ethical tensions that international health workers must navigate, operating in a context widely described as one of injustice and repeated atrocities. There is a substantial moral distress associated with trying to save lives in Gaza while being forced to remain silent or neutral on the causes of those wounds [120]. Some doctors have spoken out, writing open letters condemning the attacks on healthcare and urging colleagues abroad to not treat Gaza as just a natural disaster zone but a political atrocity [121, 122]. The U.S. medical establishment in particular was criticised for its ‘dangerous silence’ on Gaza's genocide [121, 123]. This has implications: when humanitarian professionals are constrained from advocating for political change (lest they jeopardise their operations), their missions inadvertently become part of an unsatisfactory status quo [124]. It is a limitation that many accept reluctantly as the priority is to keep helping patients, but it can gnaw at the conscience of individuals and humanitarian actors involved. For example, MSF and others have refused to accept the narrative of these hospital bombings as ‘regrettable accidents’, pointing to the systematic nature of the violence [75]. The ethical stance an organisation takes can affect its ability to operate (outspoken ones may face more difficulty getting access), which is a political trade‐off. All these factors showcase that international medical missions, no matter how skilled or well‐funded, operate in Gaza with their hands partially tied. They can staunch bleeding and deliver babies, but they cannot, by themselves, reconstruct the health system or end the suffering at its political source.

## 5 Political Determinants Hindering Health System Reconstruction

During the brief ceasefire that commenced on 19 January 2025 ‐ marked by continued bombardment and shootings by the Israeli military, and ultimately shattered by Israel's mid‐March assault that is responsible for the deadliest week on record for Palestinian children ‐ international discourse momentarily shifted to the colossal task of reconstructing Gaza's obliterated health system [125]. However, the underlying political determinants that functioned to create the fragile foundation for the accelerated destruction of the health system were overlooked and appeared set to remain intact, casting a long shadow over any rebuilding efforts [126]. In the context of Gaza, several intersecting political factors that continue to limit the prospect of effective health system recovery are outlined below.

### 5.1 Israel's Ongoing Blockade and Restrictions on Humanitarian Access

The Israeli‐Egyptian blockade of Gaza has only deepened as of early 2025, for which the transfer of food, clean water, medical equipment, pharmaceuticals, tents, and building materials, along with many other essential items, is increasingly tightly controlled. Israel's designation of many medical and reconstruction items as ‘dual‐use’ goods (supposedly susceptible to alternative use by armed groups), which includes items ranging from surgical implants, anaesthetic drugs, coffee, and insulin pens, severely hampers the replenishment of hospital supplies and the repair of essential infrastructure [16]. The Kafkaesque logistical process of trying to get CT scanners, x‐ray machines, or even concrete, to Gaza's hospitals requires Israeli military approval at multiple levels, often resulting in months of delay or outright refusal. The stranglehold maintained over the armistice line to the north and east, the sea to the west, and the border to the south, also affects staffing of the health system, as Palestinian and international medical experts cannot come and go freely for the purpose of knowledge exchange, further training, and service provision. The blockade thus reinforces the isolation of Gaza's health sector, prefiguring the limits of recovery, just as was the case before the genocide.

Associated sectors that intersect with health have also been compromised, serving to multiply the impact of manufactured deprivation. For example, direct Israeli attacks coupled with the obstruction of aid trucks containing flour have left all of Gaza's bakeries destroyed or otherwise non‐functional for prolonged periods of time. Large amounts of agricultural land has been damaged, occupied, or polluted by military ordinance [127, 128, 129, 130]. Such destruction has limited domestic food production capacity, further deepening dependency on external aid for the most basic means to sustain life [131, 132]. Without political pressure to end the blockade, any reconstruction of the health system ‐ and all other essential social systems ‐ will remain precarious and incomplete.

Israel continues to demonstrate complete control over the frequency, form, and modality of humanitarian response, limiting short term approaches and reconstruction activities that can occur in Gaza [133]. A stark example materialised in October 2024, when Israel abruptly barred at least eight international medical organisations from entering Gaza [101]. These were not political organisations but medical teams (including well‐known diaspora‐led organisations such as the Palestine American Medical Association and technical groups such as a 3D prosthetics NGO) that had provided crucial support. In a joint letter, HCWs from some of these organisations described Israel's move as a ‘punitive pattern of imposing arbitrary restrictions’ on healthcare worker entry, noting that many volunteer physicians were left in limbo or denied entry without explanation [101]. Similarly, during an uninterrupted period of 84 consecutive days in the spring of 2025, none of the six healthcare facilities in Gaza supported by MSF received deliveries of medical supplies [134]. Such actions clearly demonstrate the extent to which medical and humanitarian access is controlled by Israel, in breach of international legal obligations [135]. The effect is that needed expertise and material aid are delayed or outright blocked [136]. Additionally, the Israeli authorities have sometimes pushed their own ‘coordinated’ efforts that align with their political objectives, such as trying to route aid through military channels or promoting field hospitals that operate under their supervision [136, 137]. Humanitarian organisations have, in some cases, attempted to resist instrumentalisation by overarching political forces, but navigating this environment consumes time and resources. In essence, as long as Gaza remains under Israeli occupation, the reconstruction of its health sector is entirely subject to the political calculations of the occupying power.

### 5.2 Lack of Protection and Accountability

A fundamental barrier to meaningful recovery and just resolution is the absence of a genuine commitment to accountability for the destruction of Gaza's health system, wider exterminatory violence against Palestinians, and the derogation of Palestinian rights more broadly. Despite well‐documented evidence of war crimes and crimes against humanity in the form of the repeated targeting of HCWs, medical facilities and other health infrastructure, no effective international mechanism has functioned to prevent these attacks, or to deter subsequent attacks when genocidal intent and patterns of violence became increasingly clear.

Israel's rejection of investigations, and its allies' diplomatic shielding, mean that the perpetrator of the destruction of Gaza's health system has faced no meaningful consequences, and instead has continued to operate with near‐absolute impunity [138]. A number of humanitarian organisations have quietly expressed that pouring resources into rebuilding Al‐Shifa or Al‐Ahli Hospital, for example, feels futile if there is a risk that these facilities will be bombed again during the next offencive [139, 140]. Similar sentiments have been expressed in the context of Arab states' reconstruction plans [141, 142]. The reality that there is no guarantee of further destruction, no prospect of a binding ceasefire, and no immediate, protective intervention to safeguard protected groups and infrastructure, has tempered the willingness of donors to invest early in reconstruction efforts [143, 144].

The limitations that are inherent to repeated cycles of extreme violence and corresponding humanitarian response, demand that all organisations ‐ and particularly those that are operating in Gaza, and thus have directly witnessed the individual and population level impact of such violence ‐ advocate to break this perpetual cycle of destruction. For example, in late 2024, MSF publicly called for states to leverage influence on Israel to end its attacks, explicitly linking the ability to deliver humanitarian assistance with the pressing need for a ceasefire and respect for international law [15]. Without such political and legal safeguards, health system reconstruction efforts are likely to be short lived and ultimately unsuccessful.

### 5.3 Local Governance Issues

Rebuilding Gaza's healthcare system should not be designed, implemented or led by international actors: its success is hinged on empowered local governance. Yet Gaza's authorities are in a politically and diplomatically isolated position. The PA Ministry of Health (based in Ramallah) has had limited or no operational control in Gaza since 2007, and all indications are that the PA's potential return to administrative oversight in Gaza is heavily contested, as anti‐PA sentiment deepens [145, 146]. Meanwhile, the health authorities in Gaza who oversaw the health system during the genocide (coordinating ambulance dispatch, disease surveillance, etc.), are still not recognised as legitimate partners by many external organisations and donors. This leads to a deeply problematic and profoundly inefficient situation in which parallel health service delivery arrangements emerge. For instance, the coordination of which facilities to rebuild and in what way, may fall to the UN or other international mechanisms, rather than to the Ministry of Health, as international donors refuse to fund anything that might be seen as benefiting local governance structures. This was evident in the aftermath of previous violent escalations, and persists now with donors favouring channelling funds through UN agencies or large international NGOs [26]. As OCHA noted in 2017, no‐contact policies ‘further restricted the operational space of international NGOs in Gaza’, forcing them to sideline the local authorities [26]. The consequence is often duplication or gaps: resources gravitate towards projects that are politically ‘safe’ rather than those most needed by the population. A clinic might be rebuilt in a refugee camp (under UNRWA's mandate) while a Ministry‐run clinic in a non‐refugee area remains in ruins for lack of funding. This fragmentation undermines the possibility of cohesive health system recovery. Furthermore, Palestinian health professionals remain caught in the middle of these manufactured political divisions; many who worked for the Gaza Ministry of Health have not received regular salaries in years, and now amid the genocide and further political and economic disruption, this is likely to affect morale and risks driving the emigration of skilled workers at a time when their expertise is most needed.

### 5.4 Donor ‘Fatigue’ and Geopolitical Priorities

Reconstruction efforts in Gaza, in the wake of such extensive devastation, will require a Marshall Plan‐level influx of resources and a sustained commitment to health system strengthening [133]. Yet politically speaking, Gaza competes for attention in a world of poly‐crises. There is a palpable fatigue and wariness among some traditional donors, partly due to the politicisation of aid to Palestinians in recent years [80, 147]. Some Western governments face domestic political pressure against providing financial and other forms of support in Gaza (viewed through the lens of not ‘supporting terrorism’, among other reasons), while others channel funds primarily through substantial humanitarian operations, which treat downstream humanitarian consequences but shy away from infrastructural and other projects that imply permanence and allow for meaningful recovery [26, 147, 148, 149, 150, 151]. The end result is that appeals to the reconstruction of Gaza's health sector remain woefully underfunded relative to need [138, 152, 153]. In early 2025, a correspondence in *The Lancet* by Nasari et al. argued that the ceasefire offered a ‘critical opportunity’ to redesign Gaza's health system with a focus on resilience, but only if the international community moved beyond a fixation on short‐term aid [17]. They noted that past conflicts have shown that emergency aid alone ‘does not translate into sustained health system development and has often failed to address the root causes of health‐care instability’. Without a political shift to address those root causes, namely, the blockade, occupation, and funding constraints, international pledges risk focussing on repeated cycles of short‐term humanitarian response rather than meaningful reconstruction and recovery. The deepening challenges affecting the entry and distribution of aid do little to allay donor fatigue and scepticism that funds raised will not translate into material assistance to Palestinians in Gaza. Even prior to the destruction of the Rafah crossing, thousands of aid trucks sat idle for miles with perishables rotting under the Sinai desert sun [154]. Palestinians in Gaza have followed many lofty donor conference promises since 2006, only to see little change on the ground [152, 155, 156]. The questionable credibility of international support now poses its own challenge, as communities that have lost faith in broken promises are understandably less likely to engage in proposed rebuilding efforts [157].

## 6 Towards Accountability and Reform: A Path for Sustainable Reconstruction

For Gaza's health system to recover from the extensive devastation wrought by Israel's genocide, and for international engagement to provide meaningful rather than piecemeal and ultimately ineffective support, a major paradigm shift is required at multiple levels.

### 6.1 Accountability for Destruction

The first step towards meaningful reconstruction and recovery is meaningful procedural action to hold accountable those responsible for the decimation of health facilities, other war crimes and crimes against humanity. Calls for accountability are not a matter of retribution, but serve as a commitment to justice, deter future attacks and violations, and function to create a safer environment for recovery [158, 159]. International humanitarian law is clear that hospitals, ambulances, and health workers are all protected [160]. The pattern of strikes on Gaza's hospitals documented since 2023 is so egregious that UN experts, Amnesty International, Human Rights Watch, and many others have all called for war crimes investigations [161, 162]. These calls need to be answered, and corresponding investigations must have tangible implications. If genocide and crimes against humanity are to have meaning, they must translate into international action against Israel to halt its exterminatory violence, interventions to protect the Palestinian people, and reparatory responses for survivors. Practically, reparations in the context of Gaza's health system should require that Israel and its political allies finance and otherwise resource the reconstruction and rehabilitation of healthcare facilities. This is not unprecedented ‐ in other conflicts, aggressors have been pressed to compensate for damage and destruction [163]. Without accountability there can be no justice, and without justice there can be no complete recovery. Hospitals may be reconstructed with new equipment, but if an emboldened military can bomb again with impunity, the risk of further violence and destruction will remain. Palestinians, healthcare professionals in Gaza and worldwide, and solidarity activists elsewhere, have all demanded that the commitment to ‘never again’ be applied to attacks on healthcare [164, 165, 166]. Ensuring protection for the medical mission in Gaza is a legal and political obligation, which all states are duty bound to uphold.

### 6.2 Ending the Occupation

It is impossible to speak seriously about recovery of the health or other sectors in Gaza without addressing the occupation [167]. The occupation manifests most tangibly in the form of a state of blockade and siege, which limits the transfer of water, food, medicines, fuel, and movement [168]. No amount of external aid can offset the structural violence and health consequences of the blockade, manifesting in stunting in children, chronic traumatic stress disorders, among many other health issues. As a consequence, the foundation of any reconstruction and recovery plan must be an end to Israel's occupation and its blockade. Multiple UN agencies, ranging from UNRWA, the WHO, to OCHA have long called for this, and a growing number of medical voices have echoed these demands [169, 170]. The vision of a resilient health system in Gaza is inextricably connected to the pre‐condition that the blockade is brought to an end and Palestinians, Palestinian civil society, and Palestinian governing authority, are able to realise sovereignty and autonomy. This is required for the flourishing of Palestinian‐led service delivery, which is impossible under the conditions of occupation that restrict every aspect of Palestinian life. States and other donors can condition their post‐genocide assistance not on political reconfigurations and disarmament quotas, but on compliance with legal rulings on the illegality of Israel's occupation and imposition of apartheid conditions [171, 172]. Health system reconstruction does not just concern the reestablishment of physical structures, but rather concerns the non‐derogable and universal right to health [173]. Ending the siege is thus an act of structural prevention: mitigating against a deepening health crisis by directly addressing the conditions of manufactured scarcity [174, 175]. This is certainly a political challenge, but framing it as a health imperative has the potential to rally broader international consensus, especially given the stark evidence of blockade‐induced mortality and other negative health outcomes [176, 177, 178, 179, 180].

### 6.3 Inclusive Governance and Palestinian Leadership

Any reconstruction effort must empower Palestinian leadership rather than bypass its authority and expertise [114, 181]. This may require creative mechanisms in the interim; one proposal put forward by policy experts is the creation of a Gaza Health Reconstruction Council, which brings together representatives of the Ministry of Health in Gaza, the PA Ministry of Health, UN agencies, and key donor states, on a joint platform [182, 183]. Such a Council could coordinate rebuilding plans, ensure resources are aligned with needs, and serve as a politically recognised platform between Palestinian‐run local structures and international states and donors. Decision‐making that circumvents Gaza's health professionals should be roundly rejected. Those Palestinian healthcare workers and administrators who maintained health service provision throughout Israel's protracted genocide have invaluable expertise and knowledge, and retain ownership of their own health system [184]. International actors should purposefully shift from direct implementation to a support role as soon as is feasible, channeling funds and material support to local institutions. This aligns with the humanitarian sector's Grand Bargain commitment to ‘localization’, that is, shifting power to local and national responders: a principle frequently invoked but as yet seldom actualised in humanitarian crises [109]. In practice, this requires an increase in funding to Palestinian NGOs and professional associations that are involved in the provision of healthcare. Most foundationally it requires involving Palestinians in decision‐making, and ensuring their genuine leadership in all stages of post‐genocide recovery. Building back a health system is far from a technocratic exercise, but requires trust and ownership. Many Palestinians have lost trust in existing providers as they saw health facilities transformed into death traps, while other providers failed to speak out against atrocities committed against the Palestinian people. Encouraging Palestinians to seek care again in rebuilt facilities requires demonstrating that the situation has tangibly changed. Palestinian leadership, with meaningful community engagement, is best positioned to restore that trust [185].

### 6.4 Rejecting Parallel Structures

Humanitarian actors should deliberately align their interventions with national health policies and frameworks and transfer operational control to Palestinian authorities at the earliest opportunity. For example, if an internationally‐managed field hospital remains functional long after a genuine ceasefire is agreed, its leadership ought to be training cohorts of Palestinian healthcare workers and administrators to assume full responsibility for the facility or to integrate its services into the wider hospital network. The WHO could spearhead an initiative to bring all field hospitals under a common coordination agreement that ensures clear Ministry of Health oversight, and thus prevents the creation of permanent parallel systems and tiered service provision [186]. As noted, parallel systems may be unavoidable during the acute phase of a crisis, but in the reconstruction and recovery periods should quickly give way to a unified and coordinated system [183]. Instructive precedents to learn from are instances in which Health Clusters in other crises have worked to strengthen local health systems, wherein alignment with national health plans is key to achieving a lasting impact [187].

In Gaza, that means aligning with health sector strategies as devised by Palestinian health experts for the post‐genocide period. For example, if strategies focus on the revival of the primary healthcare system, international NGOs should resist the temptation to establish their own primary clinics and poach Palestinian healthcare workers, and instead support the refurbishment of existing clinics and support to community health workers under a common curriculum. While it is inevitable that international organisations will hire Palestinian doctors and nurses, they should do so in a way that is sensitive to the human resources for health requirements of the wider health system. Salary support schemes, wherein donors pay stipends to government‐employed healthcare workers, can help ensure that service providers are not lost entirely to the NGO and private sectors. There is precedent for donors in other post‐conflict settings to cover public health worker salaries temporarily to stabilise the workforce [188]. Gaza will likely require similar support, given the state of manufactured financial collapse and resulting salary issues. If NGOs coordinate to support such schemes rather than compete for the best‐trained Palestinian staff, this will also mitigate against a critical dimension of parallel system formation. Additionally, there is a need for clear acts of solidarity by the international medical associations and healthcare workers, in the form of partnerships with Palestinian institutions and healthcare workers that endure in the wake of the genocide. For example, twinning hospitals in Gaza with hospitals abroad can facilitate training, telemedicine support, staff exchanges, and material assistance in a structured way that strengthens capacity over time. Some of this work began during the genocide in the form telemedicine trauma support via Palestinian surgeons in exile abroad.

### 6.5 Advocacy and the Engaged Witness

Alternative approaches to international engagement must incorporate a clearer commitment to engaged advocacy. Humanitarian neutrality must not be misinterpreted as a commitment to silence in the face of extreme violence, human rights abuses, and atrocity crimes [1, 189]. The global medical community has a professional and moral obligation to speak out against violence and abuses committed against Palestinian healthcare workers and all Palestinians. Advocacy for health must be recognised as integral to the pursuit of accountability and justice in Gaza. Where health actors, NGOs, and UN agencies raise the alarm collectively ‐ as they have done at times by issuing joint statements ‐ they can galvanise political pressure while diffusing the risk of individual institutional targeting by belligerents [190, 191, 192, 193, 194]. Medical journals and associations must also recognise their role by publishing data and editorials, issuing statements, and taking principled action, in a way that centres the voices and perspectives of Palestinians, and that rejects the normalisation of Israel's rights abuses, violence, occupation and apartheid. The aim of such advocacy must be to push for wider engagement with the underlying political determinants of health: an end to Israel's occupation, its punitive blockade and repeated breaches of international law, all of which are prerequisites to meaningful recovery.

## 7 Conclusion

In Gaza, neither humanitarian efforts nor internationally‐led interventions can substitute for the sustained and collective pursuit of just political resolution for the Palestinian people. Israel's destruction of the health system, its ongoing blockade, and its repeated attacks on civilians and other protected groups, have shifted an already fragile health system into a state of deepening crisis, far beyond the remedial reach of short‐term interventions and humanitarian approaches. Although international medical deployments have provided lifesaving assistance at brief moments in time, their temporary presence and a frequent lack of partnership with local authorities, serves to reinforce a fragmented system that remains heavily dependent on external aid. In the absence of clear accountability measures imposed on Israel for its attacks on Palestinian healthcare and other crimes, and without ending Israel's occupation and ending its associated blockade, every attempt at reconstruction is destined to fail. Only a paradigm shift that prioritises political accountability, respects Palestinian leadership, and ensures structural independence and political sovereignty will allow for meaningful health system recovery in Gaza.

## Conflicts of Interest

The authors declare no conflicts of interest.

## Data Availability

Data sharing not applicable to this article as no datasets were generated or analysed during the current study.
